# Development and preliminary clinical validation of a mobile health application for pressure injury staging in ICU patients

**DOI:** 10.3389/fmed.2026.1820690

**Published:** 2026-05-28

**Authors:** Han Sheng, Chen Hu, Danying Zhang, Zhihong Zhu, Dong Xu

**Affiliations:** 1 Department of Pain, The Affiliated Hospital of Jiaxing University, Jiaxing, China; 2 Department of Endocrinology, The Affiliated Hospital of Jiaxing University, Jiaxing, China; 3 Department of Nursing, The Affiliated Hospital of Jiaxing University, Jiaxing, China; 4 Tongji Zhejiang College, Jiaxing, China

**Keywords:** ICU, mHealth, mobile health application, nursing assessment, pressure injury

## Abstract

**Background:**

Pressure injury is a common adverse event among critically ill patients and is associated with poor clinical outcomes, increased nursing workload, and substantial healthcare costs. Accurate staging of pressure injuries is essential for guiding nursing interventions; however, conventional visual assessment is highly dependent on individual experience and often shows limited consistency.

**Methods:**

A prospective cross-sectional study was conducted in the intensive care units (ICUs) of tertiary hospitals. A smartphone-based application integrating wound image acquisition and intelligent assisted staging was developed. Pressure injury images were collected by trained nurses following a standardized protocol. Each injury was independently staged by a wound, ostomy, and continence nurse (WOCN), and a consensus reference standard was established. The staging results generated by the application were compared with expert consensus using an agreement analysis.

**Results:**

A total of 52 patients with 269 pressure injuries ranging from Stage 1 to Stage 4 were included in the study. To assess the consistency of expert-based staging, the weighted kappa value was calculated. The results showed that the weighted kappa value between the two WOCNs was 0.872 (95% CI: 0.698–0.944, *p* < 0.001), indicating a high level of agreement between the expert assessments. Further comparison of the mobile health application and the expert consensus revealed that the inter-rater agreement coefficient between the iOS version of the application and expert consensus was 0.769 (95% CI: 0.632–0.906, *p* < 0.001), while the inter-rater agreement coefficient between the Android version and expert consensus was 0.821 (95% CI: 0.698–0.944, *p* < 0.001), both showing substantial agreement. The classification consistency for each stage of pressure injuries ranged from 76.9 to 84.6%. Additionally, the inter-rater agreement between the registered nurse and the mobile health application for wound length, width, and area measurements was good (range: 0.843–0.914, *p* < 0.001). The intra-rater reliability measurements for wound length, width, and area in both the iOS and Android systems were excellent (iOS range: 0.952–0.986, Android range: 0.959–0.975, *p* < 0.001).

**Conclusion:**

The application was easy to operate and was well accepted by nursing staff. This study provides preliminary evidence that a mobile health application can serve as a feasible and objective adjunct tool for pressure injury staging, supporting more standardized and consistent nursing assessments.

## 1 Introduction

Pressure injury (PI) is defined as localized damage to the skin and/or underlying soft tissue, usually occurring over a bony prominence or in association with medical devices, resulting from sustained pressure or pressure in combination with shear forces (1). It is among the most common adverse events affecting hospitalized patients worldwide (2). Critically ill patients in the ICU are at particularly high risk due to severe illness, prolonged immobility, impaired tissue perfusion, and frequent exposure to invasive medical devices, making pressure injury a persistent and significant challenge in critical care practice (3). Previous investigations have reported that the incidence of hospital-acquired pressure injuries in the ICU ranges from 16.9 to 23.8% (4), which is approximately 10 times higher than that observed in general wards (5). The development of PIs can exacerbate patients’ conditions: chronic, non-healing wounds may lead to systemic infections such as sepsis; adversely affect quality of life; prolong hospital stays; increase the risk of mortality; and substantially increase the caregiving burden on patients and their families (6–8). Prevention and early management of PIs, therefore, represent a core priority and a major challenge in intensive care nursing. Early recognition of skin and tissue damage and accurate classification of injury severity are fundamental to effective prevention strategies and timely intervention (9). PI staging serves as the essential clinical basis for selecting appropriate preventive measures, guiding wound management, and determining suitable therapeutic dressings (10). However, in routine clinical practice, PI staging is largely dependent on visual skin assessment and individual clinical judgment, which introduces a high degree of subjectivity (11). As a result, inter-rater consistency is often limited, particularly in the identification of early-stage PIs. Previous studies have demonstrated that the accuracy of PI staging by nurses in routine real-world clinical settings ranges from only 30 to 70% (12–14). Inaccurate staging may delay appropriate intervention, lead to suboptimal wound management, and ultimately compromise patient outcomes and nursing care quality (14). Studies have shown that the rate of reduction in PI size is closely associated with wound healing, and this indicator can be used to predict the healing process of wounds (15, 16). Therefore, measurement of the PI area is of great importance in clinical practice, as it not only facilitates dynamic monitoring of the healing process of PIs but also provides objective feedback on the effectiveness of different treatment interventions.

In recent years, with the rapid development of mobile health and artificial intelligence technologies, smartphone-based wound image acquisition and digital analysis tools have increasingly been explored to support PI assessment (17). Existing studies have shown that mobile applications can assist with wound measurement, image documentation, and automated classification and have demonstrated promising reliability in specific populations, such as patients with spinal cord injury or in home-based and caregiver-supported care contexts (18). Other recent studies have focused primarily on the development of artificial intelligence models for PI stage classification with high algorithmic accuracy (19). Despite these advances, significant gaps remain in the development and clinical implementation of wound care applications. First, many existing applications primarily target wound size measurement or algorithm development, with limited emphasis on nursing-oriented staging workflows and clinical decision support (18). Second, a majority of the validation studies have been conducted in rehabilitation, community, or home care settings, whereas evidence from intensive care environments—where PI risk is highest and assessment conditions are most complex—remains scarce (17). Third, several studies focus on technical performance under controlled conditions but lack clinical validation against wound care specialists in real-world bedside scenarios, limiting their immediate applicability to routine nursing practice (20).

Given these limitations, there is a clear need for a clinically oriented mobile assessment tool that is specifically developed and validated for intensive care settings and that emphasizes consistency with expert nursing judgment and practical feasibility in routine care. Accordingly, this study aimed to develop a mobile health application for PI staging based on wound image analysis and to preliminarily evaluate its feasibility and agreement with wound care specialist assessments among intensive care unit patients.

## 2 Materials and methods

### 2.1 Participants and setting

**Figure 1:**
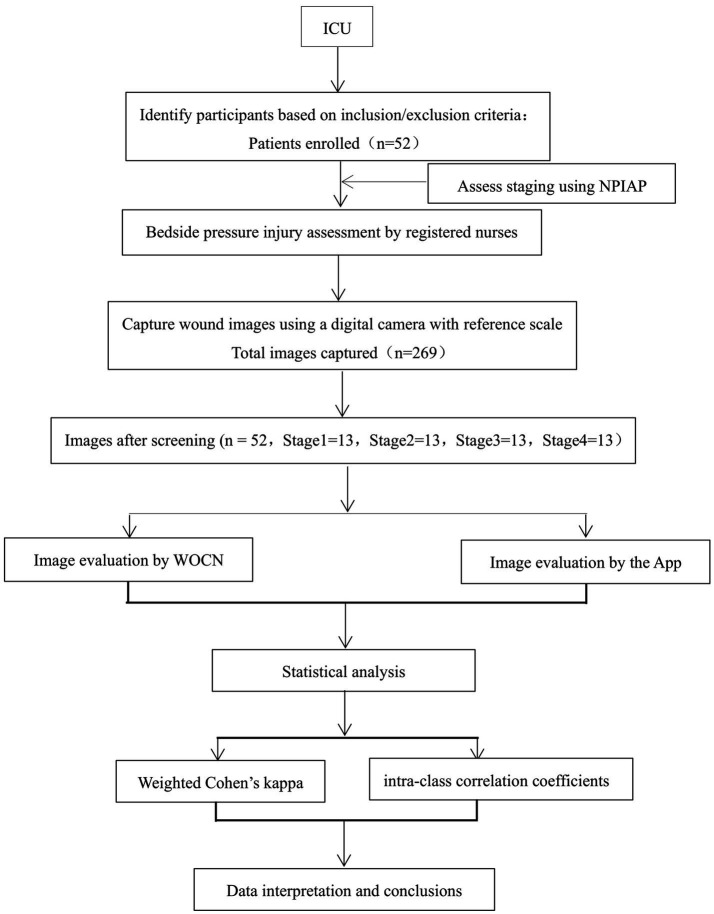
Study workflow and image analysis process.

A prospective cross-sectional study was conducted between January and December 2024 in the ICUs of tertiary hospitals in Jiaxing, China. This study aimed to develop and preliminarily validate a mobile health application for PI staging in critically ill patients. During the study period, all patients admitted to the ICU were screened. Registered nurses (RNs) performed an initial screening for suspected PIs as part of routine nursing assessment. Patients who met the inclusion and exclusion criteria and received confirmed PI staging were enrolled. The overall study workflow and image analysis process are illustrated in Figure 1. Eligible participants were patients who met the following inclusion criteria: (1) a confirmed diagnosis of Stage 1 to Stage 4 PI according to the National Pressure Injury Advisory Panel (NPIAP) classification (Table 1), as assessed by a wound, ostomy, and continence nurse (WOCN); and (2) full exposure of the PI site allowing adequate image acquisition. Patients were excluded if they met any of the following criteria: (1) skin lesions not attributable to PI, such as burns or inflammatory skin diseases; (2) incomplete clinical data; and (3) wound areas (>100 cm^2^), which could not be fully captured within a single standardized image. After enrollment, demographic and basic clinical data, including age and sex, were extracted from the hospital electronic medical record system. Each eligible PI was subsequently included in the standardized image acquisition and staging procedure.

**Table 1:** National pressure injury advisory panel classification criteria for pressure injury staging.

**Stage**	**Characteristic**	**Image**	**Treatment**
Stage 1	The skin remains intact with non-blanchable erythema in a localized area, typically over a bony prominence.	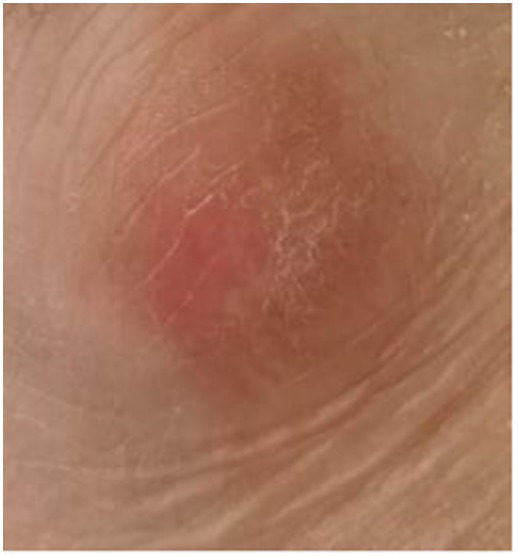	Pressure relief Adjust the position Maintain skin hygiene
Stage 2	Partial-thickness skin loss presenting as a shallow, open ulcer with a pink wound bed, without slough; it may also appear as an intact or ruptured serum-filled blister.	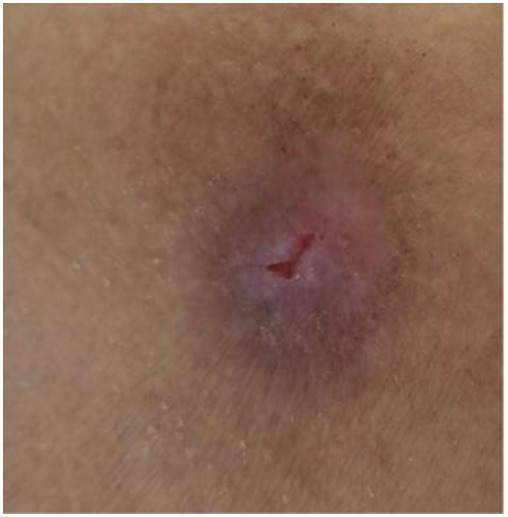	Pressure relief Adjust the position Maintenance of a moist wound environment Use of protective dressings
Stage 3	Full-thickness skin loss with visible subcutaneous fat but without exposure of bone, tendon, or muscle. The depth of tissue damage varies by anatomical location, and areas with significant adipose tissue may develop deeper wounds.	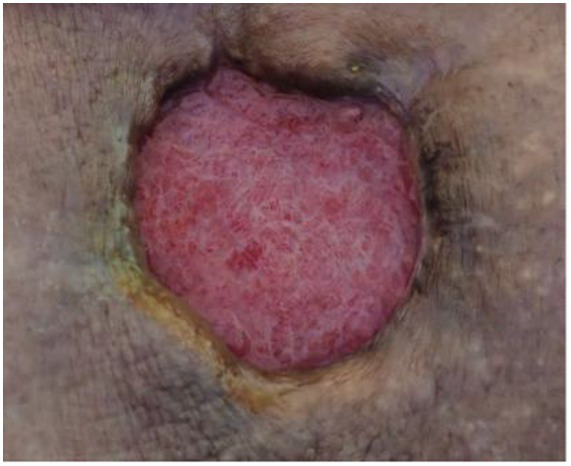	Debridement Pressure relief Adjust the position
Stage 4	Full-thickness skin and tissue loss with exposed bone, tendon, or muscle. Slough and/or eschar may be present, and undermining or tunneling is often observed. The depth of the wound varies depending on anatomical location.	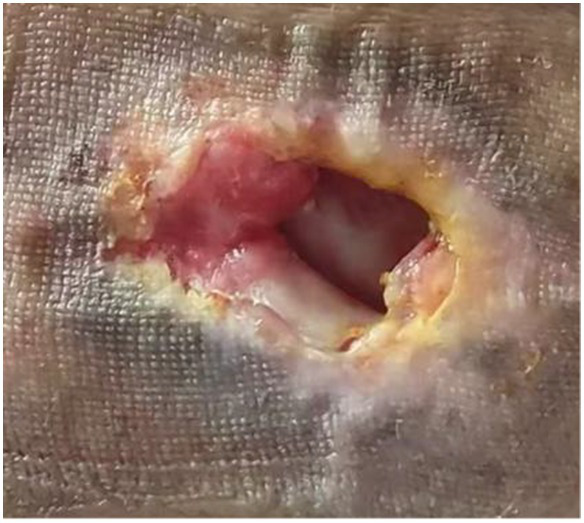	Multidisciplinary management Infection control Surgical intervention when necessary

### 2.2 Selection and optimization of the YOLOv8s model

This study used a dataset of PI images collected clinically, comprising a total of 366 images. All images were annotated by WOCNs according to the NPIAP standards and categorized into four distinct stages of pressure injuries: 72 images for Stage 1, 135 images for Stage 2, 71 images for Stage 3, and 88 images for Stage 4. The dataset was randomly split into training and validation sets at an 8:2 ratio. The resolution of all PI images was standardized to 640 × 640 pixels. To enhance the model’s generalization ability and mitigate the issue of limited training data, a series of data augmentation strategies were applied, including random cropping, random horizontal flipping, random rotation, and color jittering (brightness, contrast, and saturation).

During the annotation process, uniformly trained nurses used the LabelMe (v5.3.1) tool to perform rectangle-based annotations on the PI images across all four stages. The annotations were recorded in .txt files detailing the position and class information for each image. Subsequently, JSON files formatted using LabelMe were converted to the YOLO format to facilitate training of the deep learning model.

We embedded the Spatial and Channel Synergistic Attention (SCSA) mechanism into the Crossover Convolutional Fusion (C2f) module of YOLOv8s to improve the model’s accuracy in recognizing PIs in complex environments. The improved YOLOv8s model achieved the following accuracy for PI staging: 89.3% for Stage 1, 84.3% for Stage 2, 73.2% for Stage 3, and 100% for Stage 4. The model’s recall rate was 83%, with a mean average precision (mAP50) of 92.0%, a mAP50:95 of 65.2%, and an F1 score of 0.85.

### 2.3 Development and implementation of the mobile health application

To address the high subjectivity and inter-nurse variability in pressure injury staging, particularly in time-constrained intensive care environments, we developed a smartphone-based, non-contact mobile health application compatible with both iOS and Android platforms. The application integrates wound image acquisition, intelligent assisted staging, wound area calculation, and result storage to support pressure injury assessment in clinical nursing practice.

**Figure 2:**
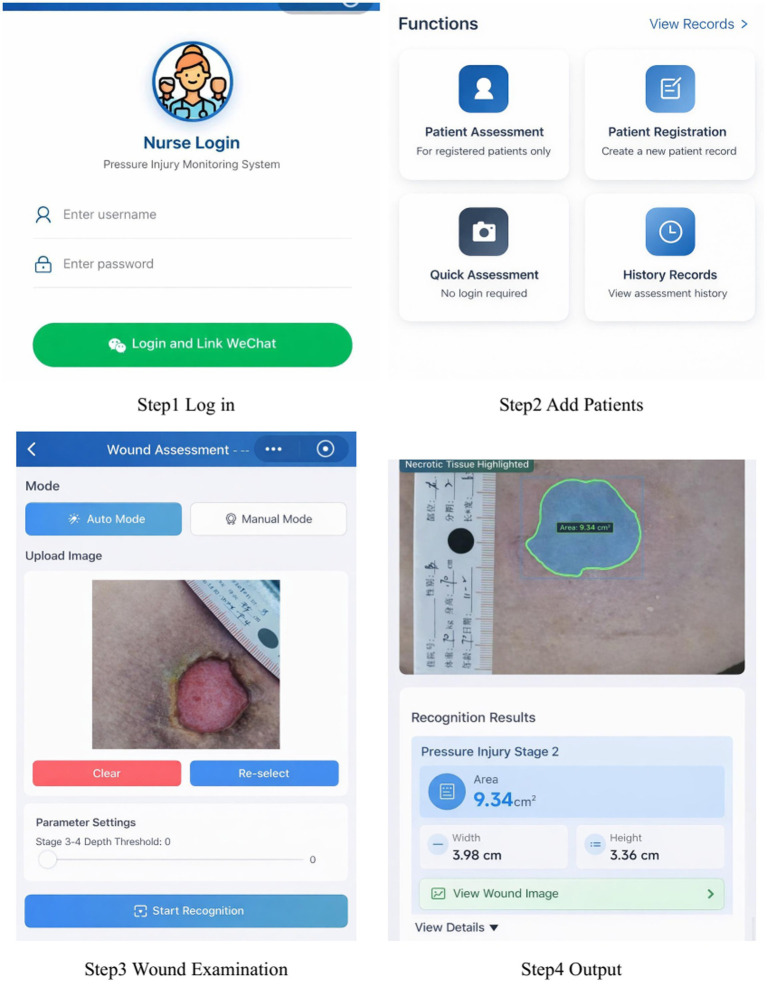
Workflow of the mobile health application.

In the mobile health application, the YOLOv8s model is embedded to process images captured by the smartphone’s camera in real time, automatically performing staging analysis and providing suggestions. To ensure the reliability of the application in clinical settings, the development process involved iterative testing by the research team and software engineers to optimize the image acquisition procedure, system stability, data storage, and user interface design. Particular attention was given to usability in intensive care environments, along with enhanced data security and privacy protection. The workflow for using the mobile application is shown in Figure 2.

The application requires user authentication during login and only allows authorized users to access patient data and history records. The linkage with external platforms, such as WeChat, is limited to providing user login and information sharing functions, with all data exchanges encrypted. The application will not share patients’ private data or wound images with external platforms, ensuring strict protection of patient privacy.

### 2.4 Image acquisition and staging procedure

Pressure injury images were collected by trained intensive care nurses following a standardized protocol. Before image acquisition, patients were positioned appropriately according to their clinical condition to ensure full exposure of the target area while maintaining safety and privacy. Images were captured using smartphone cameras at a distance of approximately 40–65 cm, under different lighting conditions, and from multiple angles. Backgrounds were kept clean and uniform to minimize visual interference. For each PI, RNs photographed the wound according to standard procedures. During image acquisition, a physical ruler was placed adjacent to the wound to provide scale calibration for subsequent automated measurement. After image capture, the mobile health application automatically identified the wound boundaries and calculated the wound’s length (the longest axis), width (the longest axis perpendicular to the length), and estimated area based on the calibrated image dimensions. For each PI, the mobile health application was used to generate an automated staging result. Meanwhile, two WOCNs independently staged each injury based on the same images using visual assessment. When discrepancies occurred, a third specialist conducted an additional evaluation to establish a final consensus reference standard. The staging results generated by the mobile application and those determined by the WOCNs were systematically recorded for subsequent agreement analysis.

### 2.5 Image analysis process

The mobile health application uses a deep learning-based image analysis framework derived from the YOLOv8s architecture for automated pressure injury staging and wound measurement. After image upload, the wound region was first detected and separated from the surrounding healthy skin tissue. The model then performed multi-scale feature extraction to analyze wound-related visual characteristics. The main image features considered by the system include color distribution, tissue texture, wound boundary morphology, geometric dimensions, and relative depth-related visual cues. In addition, when visible, the presence of exposed subcutaneous tissue or deeper anatomical structures was incorporated into the classification process.

These extracted features were integrated to classify pressure injuries according to the established clinical staging definitions. Specifically, Stage 1 injuries were characterized by intact skin with persistent erythema or discoloration; Stage 2 injuries by partial-thickness skin loss, abrasion, or blistering; Stage 3 injuries by full-thickness skin loss with visible adipose tissue; and Stage 4 injuries by deeper tissue loss with exposed fascia, muscle, tendon, or bone. To improve discrimination between adjacent stages, an attention-enhanced feature fusion module was incorporated to strengthen the recognition of subtle visual differences.

### 2.6 Reference standard

The consensus staging determined by WOCNs served as the reference standard. Agreement between the two primary specialists was first evaluated. If both specialists assigned the same stage, this result was accepted as the final classification. If disagreement occurred, a third specialist reviewed the images and adjudicated the final stage. This consensus process was adopted to minimize subjectivity and to provide a clinically robust reference for validation.

### 2.7 Sample size calculation

As a preliminary validation study, the sample size was determined pragmatically based on the availability of eligible cases during the study period and prior methodological studies evaluating diagnostic agreement and classification performance. From the eligible cases, 52 representative pressure injury images covering Stages 1 to 4 were selected through random sampling. This validation set was considered sufficient for exploratory agreement analyses.

### 2.8 Statistical analysis

All statistical analyses were performed using *SPSS* software (version 26.0). Continuous variables were summarized using means and standard deviations or medians and interquartile ranges, as appropriate. Categorical variables were described as frequencies and percentages. Weighted Cohen’s kappa coefficient was used to assess the inter-rater agreement between the two WOCNs and to assess agreement between the mobile health application and the specialist consensus staging. For the length, width, and area of PI, intra-class correlation coefficients (ICCs) were used to analyze the inter-rater reliability between measurements obtained by the responsible nurses and those obtained using a mobile application running on iOS or Android systems, based on a two-way mixed-effects model with absolute agreement and single measure. Intra-rater reliability for length, width, and area between images taken by the mobile health application on the same device (for both iOS and Android systems) was also analyzed using a two-way mixed-effects model, absolute agreement, and single measure. Confusion matrices were constructed to describe classification distributions across different stages, and stage-specific accuracy was calculated using the specialist consensus as the reference standard. All statistical tests were two-sided, and a *p*-value of <0.05 was considered statistically significant.

The ICC criteria adopted in this study were as follows: ICC values range from 0 to 1, with ICC of <0.5 indicating poor reliability, 0.5–0.75 indicating moderate reliability, 0.75–0.9 indicating good reliability, and ICC of > 0.9 indicating excellent reliability (21).

The kappa criteria adopted in this study were as follows: the kappa coefficient ranges from −1 to 1, with larger values indicating greater agreement between the two results. A kappa value of ≥ 0.75 indicates good agreement, whereas a value of < 0.4 indicates poor agreement (22).

## 3 Results

### 3.1 Participant characteristics

A total of 52 patients with PIs admitted to the intensive care units were included in the final analysis. The mean age of the patients was 72.6 years, and 34 of them were male patients (65.4%). The mean body mass index was 24.1 kg/m^2^. During the image acquisition process, a total of 269 wound images were collected from these patients for measurement reliability analyses. For staging validation analyses, one representative PI image was selected from each patient, resulting in an independent validation dataset of 52 images. According to the expert consensus staging of the validation dataset, 13 images were classified as Stage 1, 13 as Stage 2, 13 as Stage 3, and 13 as Stage 4. During screening, two individuals were identified with unstageable or deep tissue pressure injuries; however, as these conditions did not meet the eligibility criteria, they were excluded from the study. Additionally, one patient was excluded due to a pressure injury wound area exceeding 100 cm^2^.

### 3.2 Inter-rater agreement between WOCNs

To evaluate the reliability of expert-based PI staging, 52 representative PI images were independently assessed by two WOCNs using an online questionnaire platform. Inter-rater agreement was examined using the weighted Cohen’s kappa statistic. The weighted Cohen’s kappa value was 0.872 (95% CI: 0.698–0.944, *p* < 0.001), indicating a high level of inter-rater agreement in image-based PI staging.

Using the consensus staging results of the WOCNs as the reference standard, agreement between the mobile health application and expert assessment was further evaluated. The inter-rater agreement coefficient between the iOS version of the application and the expert consensus was 0.769 (95% CI: 0.632–0.906, *p* < 0.001), while the coefficient between the Android version and the expert consensus was 0.821 (95% CI: 0.698–0.944, *p* < 0.001), both demonstrating substantial agreement. Confusion matrix analysis showed that the mobile health application achieved good classification performance across all PI stages (Table 2). The classification accuracies for Stage I, Stage II, Stage III, and Stage IV PIs were 84.6, 76.9, 76.9, and 84.6%, respectively.

**Table 2:** Agreement between the mobile health application and WOCNs in pressure injury staging.

Predicted	Stage	**Ratings by WOCNs (Actual)**			
		**Stage 1**	**Stage 2**	**Stage 3**	**Stage 4**
Ratings by mobile medical application (predicted)	Stage 1	84.6 (11/13)	7.7 (1/13)	7.1 (1/13)	0.0
	Stage 2	7.7 (1/13)	76.9 (10/13)	7.7 (1/13)	7.7 (1/13)
	Stage 3	7.7 (1/13)	15.4 (2/13)	76.9 (10/13)	7.7 (1/13)
	Stage 4	0.0	0	7.7 (1/13)	84.6 (11/13)

### 3.3 Inter-rater reliability of pressure injury measurements

Good inter-rater reliability was observed between the RNs and the mobile health application on both iOS and Android devices for PI length, width, and area measurements (iOS: ICC range: 0.843–0.900, *p* < 0.001; Android: ICC range: 0.896–0.914, *p* < 0.001) (Tables 3, 4).

**Table 3:** Inter-rater reliability between the RN and iOS device for the corresponding image.

Assessment source	**Measurements, mean±SD**			**Inter-rater reliability (95*%CI*)**			***P*-value**		
	**Length**	**Width**	**Area**	**Length**	**Width**	**Area**	**Length**	**Width**	**Area**
iOS	2.73 ± 0.54	2.04 ± 0.60	5.74 ± 2.43	0.843	0.861	0.900	<0.001	<0.001	<0.001
Nurse	2.84 ± 0.56	2.07 ± 0.60	6.09 ± 2.73	0.727–0.910	0.769–0.918	0.826–0.943			

Length and width are expressed in cm; area is expressed in cm^2^; SD: standard deviation; 95%CI: 95% confidence interval.

**Table 4:** Inter-rater reliability between the RN and Android device for the corresponding image.

Assessment source	**Measurements, mean ± SD**			**Inter-rater reliability (95*%CI*)**			***P*-value**		
	**Length**	**Width**	**Area**	**Length**	**Width**	**Area**	**Length**	**Width**	**Area**
Android	2.75 ± 0.53	1.98 ± 0.56	5.61 ± 2.37	0.903	0.896	0.914	<0.001	<0.001	<0.001
Nurse	2.84 ± 0.56	2.07 ± 0.60	6.09 ± 2.73	0.821–0.946	0.814–0.941	0.816–0.956			

Length and width are expressed in cm; area is expressed in cm^2^; SD: standard deviation; 95% CI: 95% confidence interval.

When the mobile health application was used on iOS and Android platforms to measure three different images of the same PI, excellent intra-rater reliability was observed for length, width, and area measurements. The ICC values ranged from 0.952 to 0.986 on the iOS platform and from 0.959 to 0.975 on the Android platform (*p* < 0.001) (Table 5).

**Table 5:** Intra-rater reliability within the same device (iOS or Android) on three different images obtained from the same PI.

Assessment source	**Measurements, mean ± SD**														
	**Image 1**			**Image 2**			**Image 3**			**Inter-rater reliability (95%CI)**			***P*-value**		
	**Length**	**Width**	**Area**	**Length**	**Width**	**Area**	**Length**	**Width**	**Area**	**Length**	**Width**	**Area**	**Length**	**Width**	**Area**
Android	2.75 ± 0.53	1.97 ± 0.56	5.61 ± 2.37	2.83 ± 0.56	1.99 ± 0.56	5.82 ± 2.46	2.81 ± 0.57	2.01 ± 0.55	5.82 ± 2.42	0.959(0.933 ~ 0.976)	0.975(0.961 ~ 0.985)	0.975(0.960 ~ 0.985)	<0.001	<0.001	<0.001
iOS	2.73 ± 0.54	2.04 ± 0.60	5.73 ± 2.43	2.83 ± 0.56	2.01 ± 0.58	5.84 ± 2.42	2.87 ± 0.53	2.02 ± 0.60	5.96 ± 2.44	0.951(0.886 ~ 0.976)	0.986(0.978 ~ 0.992)	0.984(0.973 ~ 0.991)	<0.001	<0.001	<0.001

## 4 Discussion

This study was conducted in an intensive care clinical setting to evaluate the assessment performance of a self-developed mobile health application for PI staging. The results demonstrated good agreement between the staging outcomes generated by the application and those determined by wound, ostomy, and continence nurses, indicating that image-based digital assessment approaches can provide stable and reliable support for PI staging. These findings are consistent with previous studies reporting that mobile- and artificial intelligence-assisted tools can achieve good reliability and accuracy in PI or wound assessment, including mobile applications designed for PI care and support and image-based classification systems for PI staging (17, 23, 24). Similar to these investigations, the present study supports the feasibility of using smartphone-based image analysis to assist wound evaluation. Importantly, our findings extend existing evidence by demonstrating substantial agreement between mobile-assisted staging and expert assessment in ICU patients, a population characterized by higher PI risk, more complex wound presentations, and greater assessment challenges.

In this study, both iOS and Android devices demonstrated good inter-rater agreement. Although subtle performance differences were observed between the two devices, the Android version slightly outperformed the iOS version in terms of inter-rater consistency; both platforms were able to provide accurate classification results across different stages of pressure injury (PI). Therefore, despite the minor differences, both devices exhibited reliable consistency, effectively supporting wound assessment and providing valuable assistance in clinical practice.

However, due to the hardware and operating system differences between iOS and Android devices, the performance of the application may vary across different devices. These differences could stem from variations in image processing frameworks, hardware acceleration capabilities, camera quality, and other factors, which may affect the accuracy and speed of image analysis. Consequently, the results of this study may be influenced by the type and configuration of the device. Future research should explore ways to optimize the application to reduce device dependency and enhance performance consistency across different platforms.

Accurate PI staging is a fundamental prerequisite for objectively describing wound characteristics, guiding clinical decision-making, and formulating appropriate nursing interventions (13). However, traditional staging methods rely heavily on visual inspection and individual clinical experience, which lack objective quantitative support and are susceptible to inter-rater variability and observer bias (25). Previous research has shown that visual skin assessment often exhibits relatively low accuracy, particularly in the identification of Stage 1–2 PIs (11, 26). Moreover, differences in clinical experience and training across hospital departments can result in inconsistent staging standards, limiting the use of PI staging as a unified indicator for nursing quality evaluation and interdepartmental comparison. In such circumstances, additional evaluation by wound care specialists is frequently required, increasing the assessment burden and potentially delaying timely intervention (3, 18). Conversely, reliance solely on individual clinical judgment may result in staging bias, potentially compromising treatment selection and delaying wound healing (25). In response to these challenges, including observer bias, subjectivity in assessment, and a lack of standardized criteria across departments, this study designed and implemented a mobile health application for PI staging, aiming to provide more standardized and objective support for clinical assessment.

In this study, the staging accuracy for Stage 1 and Stage 4 pressure injuries (PIs) was relatively high, while the accuracy for Stage 2 and Stage 3 was slightly lower. This could be due to several factors: Stage 2 PIs are shallow, involving only part of the dermis, with the wound presenting in a pink or red color, which is similar to the non-blanching erythema observed in Stage 1 PIs, potentially causing the mobile health program to misclassify some Stage 2 PIs as Stage 1. For Stage 3 PIs, the damage is more severe, often exposing fatty tissue, and may include granulation tissue or rolled edges. When Stage 3 PIs lack necrosis or eschar, they might display a color characteristic similar to Stage 2 PIs, leading the program to misclassify some Stage 2 PIs as Stage 3. Additionally, in Stage 3 PIs, the presence of necrosis or eschar can cause the wound to appear yellow or black, resembling Stage 4 PIs, which could pose a challenge for the mobile health program to accurately classify them. Furthermore, factors such as lighting conditions, angle of capture, and wound clarity may also influence the accuracy of staging. These issues reflect similar challenges that clinical nurses face when staging PIs in practice, illustrating some of the obstacles the mobile health program encounters in real-world applications.

In recent years, the application of mobile health and artificial intelligence technologies in PI assessment has expanded rapidly (17). Existing studies have shown that digital tools can assist in wound image documentation, automated measurement, and PI classification (25). Some investigations have primarily focused on technical performance, such as wound area calculation or algorithm development using retrospective image datasets, while other studies have explored mobile applications in specific populations, such as individuals with spinal cord injury or in home-based and caregiver-supported care settings (27). These studies have shown the technical feasibility and potential value of image-based assessment tools. However, many of them have emphasized algorithmic performance or measurement accuracy, with relatively limited attention to nursing-oriented staging workflows, expert consensus validation, and feasibility in high-acuity hospital environments (28, 29). Compared with these previous studies, this research contributes additional clinically relevant evidence in several important aspects. First, this study specifically targeted intensive care unit patients, a population that has been underrepresented in prior mobile-assisted staging research despite their markedly higher PI risk and assessment complexity. Second, rather than focusing solely on algorithm accuracy, this application was designed from a nursing assessment perspective, with PI staging as the core clinical function and integration into routine bedside workflows. Third, the application was validated against wound care specialists’ judgments in a real-world clinical context, demonstrating substantial agreement and supporting its practical applicability for routine nursing practice.

With the continuous advancement of digital health technologies, image-based PI assessment tools may have broader applicability beyond the ICU (3). Future research should further explore the use of this mobile application in long-term care settings, such as nursing homes. Older adults in long-term care facilities have a high prevalence of PIs, and care staff often have diverse professional backgrounds and varying levels of wound assessment experience (30). Compared with intensive care nurses, long-term care personnel frequently lack standardized training in systematic staging and evaluation (31). In such contexts, image-based mobile assessment tools may provide intuitive and accessible support, enhance the recognition of staging characteristics, and facilitate the earlier identification and standardized management of PIs. Overall, this study provides preliminary evidence that a clinically oriented mobile health application can support accurate and consistent PI staging in intensive care settings. By integrating image-based analysis into routine nursing assessments, such tools may help to improve the objectivity, standardization, and efficiency of PI management and support more consistent nursing decision-making in complex clinical environments.

## 5 Limitations and future directions

This study has several limitations. First, due to its single-center design, the sample size was relatively small and did not include cases of unstageable PIs or deep tissue PIs, which may affect the stability and generalizability of the findings. Second, the study did not evaluate the impact of skin type on the accuracy of the application. Skin type may influence the accuracy of PI identification, making this a potential limitation. Additionally, another limitation of this study is that the evaluation criteria primarily rely on expert image interpretation rather than bedside clinical examination. Although WOCNs staged the injuries based on the same images and reached consensus when necessary, this approach does not fully account for clinical information that requires tactile examination or patient-reported data, such as pain intensity, local temperature changes, and tissue hardness. These factors are critical for the accuracy and comprehensiveness of PI staging, particularly when assessing Stage 1 and deeper tissue injuries. Furthermore, the current version of the application lacks automatic image quality detection and real-time reminder functions, indicating that users should manually ensure that the image quality meets the minimum requirements; therefore, the performance of the application may be influenced by practical factors during image acquisition, including lighting conditions, shooting angle, camera resolution, image clarity, and inter-device variability across smartphones (32, 33). The absence of this functionality is another limitation of this study. Finally, this study did not formally record or measure the time spent using the application, thereby preventing an evaluation of its specific impact on workflow efficiency. Additionally, no formal data collection on usability or user satisfaction was conducted, indicating that the feasibility and acceptability of the application have not been validated.

In light of the limitations of this study, future research should focus on the following areas. First, multicenter studies should be conducted to increase the sample size and include more cases of deep tissue PIs and unstageable PIs, thereby improving the stability and generalizability of the findings. Additionally, since skin type may influence the accuracy of PI identification, future studies should assess the impact of different skin types on the performance of the application, thereby enhancing its applicability and accuracy. Furthermore, to optimize the accuracy of pressure injury staging, future research should encourage clinical experts to incorporate tactile examination and patient-reported clinical information—such as pain intensity, local temperature changes, and tissue hardness—in staging decisions within real clinical environments, rather than relying solely on image analysis. These factors are crucial for assessing Stage 1 and deeper tissue PIs. In terms of functional improvements for the application, future versions should incorporate automatic image quality detection and real-time alert features to reduce human intervention, develop standardized operating procedures, and ensure that image quality meets minimum requirements, thereby enhancing the accuracy of image analysis and improving the user experience (32, 34). Finally, future studies should formally record and measure the time spent using the application to assess its specific impact on workflow efficiency, quantify its effectiveness in improving efficiency, and conduct formal usability testing and user satisfaction surveys to evaluate the feasibility and acceptability of the application in clinical practice, providing a basis for its further promotion and application. With these improvements, future research is expected to further validate the effectiveness of the application in PI identification and management and enhance its potential for clinical application.

## 6 Implications for nursing practice

The mobile health application developed in this study has several important implications for nursing practice, particularly in high-risk and high-intensity care environments such as intensive care units. First, by providing image-based and intelligent assisted staging of PIs, the application may support nurses in performing more objective and consistent wound assessments, reducing reliance on subjective visual judgment and individual experience. This may be especially valuable for less experienced nurses, helping to narrow variability in staging accuracy and enhance overall assessment quality. Second, the application has the potential to facilitate the earlier identification of PIs and more timely clinical decision-making. Standardized staging support may enable nurses to recognize subtle tissue changes, initiate appropriate preventive measures, and select suitable wound management strategies more efficiently, thereby contributing to improved continuity and precision of nursing care. Third, the non-contact, smartphone-based design allows bedside assessment without direct wound contact, which may reduce the risk of cross-infection and support safe and efficient clinical workflows. The integration of image acquisition and digital documentation may also promote more systematic wound monitoring, support communication among multidisciplinary teams, and facilitate continuity of care.

Finally, in addition to intensive care settings, this mobile assessment tool may be applicable in other nursing contexts with high PI risk, such as long-term care facilities, rehabilitation units, and community-based care. By providing accessible and user-friendly decision support, such tools may contribute to strengthening PI prevention and management capacity across different levels of nursing practice.

## 7 Conclusion

This study evaluated the performance of a mobile health application for PI assessment, focusing on staging judgment and wound measurement. The findings demonstrated that the application achieved good intra-rater and inter-rater reliability across both iOS and Android platforms and showed substantial agreement with conventional visual assessment. These results indicate that the mobile health application may serve as a feasible and reliable adjunct tool for PI nursing assessment, supporting more objective and standardized evaluation in clinical practice. Future studies are warranted to further validate its performance in larger and more diverse populations and to explore its applicability across different care settings.

## Data Availability

The original contributions presented in the study are included in the article/supplementary material; further inquiries can be directed to the corresponding author/s.
